# Remote Measurement in Rheumatoid Arthritis: Qualitative Analysis of Patient Perspectives

**DOI:** 10.2196/22473

**Published:** 2021-03-09

**Authors:** Katie M White, Alina Ivan, Ruth Williams, James B Galloway, Sam Norton, Faith Matcham

**Affiliations:** 1 Department of Psychological Medicine Institute of Psychiatry, Psychology and Neuroscience King's College London London United Kingdom; 2 Department of Academic Rheumatology Faculty of Life Sciences and Medicine King's College London London United Kingdom; 3 The Centre for Rheumatic Diseases Faculty of Life Sciences and Medicine King's College London London United Kingdom; 4 Department of Psychology Institute of Psychiatry, Psychology and Neuroscience King's College London London United Kingdom

**Keywords:** rheumatoid arthritis, remote measurement technologies, symptom assessment, disease management, smartphone, qualitative research, mobile phone

## Abstract

**Background:**

Rheumatoid arthritis (RA) is characterized by recurrent fluctuations in symptoms such as joint pain, swelling, and stiffness. Remote measurement technologies (RMTs) offer the opportunity to track symptoms continuously and in real time; therefore, they may provide a more accurate picture of RA disease activity as a complement to prescheduled general practitioner appointments. Previous research has shown patient interest in remote symptom tracking in RA and has provided evidence for its clinical validity. However, there is a lack of co-design in the current development of systems, and the features of RMTs that best promote optimal engagement remain unclear.

**Objective:**

This study represents the first in a series of work that aims to develop a multiparametric RMT system for symptom tracking in RA. The objective of this study is to determine the important outcomes for disease management in patients with RA and how these can be best captured via remote measurement.

**Methods:**

A total of 9 patients (aged 23-77 years; mean 55.78, SD 17.54) with RA were recruited from King’s College Hospital to participate in two semistructured focus groups. Both focus group discussions were conducted by a facilitator and a lived-experience researcher. The sessions were recorded, transcribed, independently coded, and analyzed for themes.

**Results:**

Thematic analysis identified a total of four overarching themes: important symptoms and outcomes in RA, management of RA symptoms, views on the current health care system, and views on the use of RMTs in RA. Mobility and pain were key symptoms to consider for symptom tracking as well as symptom triggers. There is a general consensus that the ability to track fluctuations and transmit such data to clinicians would aid in individual symptom management and the effectiveness of clinical care. Suggestions for visually capturing symptom fluctuations in an app were proposed.

**Conclusions:**

The findings support previous work on the acceptability of RMT with RA disease management and address key outcomes for integration into a remote monitoring system for RA self-management and clinical care. Clear recommendations for RMT design are proposed. Future work will aim to take these recommendations into a user testing phase.

## Introduction

### Background

Rheumatoid arthritis (RA) is an autoimmune disease that affects approximately 1% of the adult population in the United Kingdom [1]. Its primary symptoms are recurrent joint pain, swelling, stiffness, and deformities, contributing to fatigue, reduced ability to function [2], increased prevalence of depression [3], reduced quality of life [4], and premature mortality [5]. Symptoms vary from day to day, and disease progression is unpredictable [6]. Currently, clinical status is monitored by regular clinical appointments at fixed intervals, typically every 6 months. As symptom severity fluctuates between visits, appointments may not capture the critical time points of symptom exacerbation.

Remote measurement technologies (RMTs), including smartphone apps and wearable devices, have recently emerged as useful tools for supporting health management [7-9]. Health tracking apps enable patients to actively log changes in symptoms as well as to collect passive data from built-in smartphone sensors or wearable devices. RMT offers opportunities to track symptom severity continuously and in real time, allowing the collection of rich amounts of data in naturalistic settings and overcoming difficulties in exploring symptoms during time-limited appointments [10]. There is growing evidence to support the cost-effectiveness of mobile health interventions. A number of studies have shown positive outcomes, including improved attendance rates at health promotion centers and medication adherence, as well as positive costing outcomes on economic evaluators (eg, a score of 79.6% on the Consolidated Health Economic Evaluation Reporting Standards checklist) [11].

There is a huge appetite for the integration of RMTs into clinical care for RA. A recent systematic review reported the availability of 19 Android or iOS apps for symptom measurement in RA, representing a range of self-reported and passively collected features [12]. Along with this ambition is a growing body of evidence examining engagement with RMT for symptom tracking; the concept of RMT has a good level of face validity, with an estimated 86% of patients with RA reporting a willingness and interest in using apps for symptom monitoring [13].

Despite this ambition, evidence suggests huge variability in engagement with RMT, with adherence levels ranging between 11% and 65%, depending on the requirements for the patients, burden of questionnaire completion, and length of follow-up [14,15]. Barriers for engagement are extensive and specific to the individual; systematic review evidence suggests that perceived clinical value, symptom severity, and convenience are key drivers of uptake [16].

When developed in close collaboration with patients and clinicians, the use of apps in clinical care may positively affect the health outcomes of patients with RA. Co-design involves target end users working with researchers through development, pilot testing, and dissemination [17]. The REMORA (Remote Monitoring of Rheumatoid Arthritis) project, which carefully co-designed a symptom measurement app with patients, has demonstrated excellent levels of patient engagement and has identified temporal changes that might have been previously missed by consultants [18]. In another study, patients with RA who tracked their disease through validated questionnaires and digitally recorded joint counts better adhered to medication, better managed activities of daily living, and reported less worry about the future [19]. Incorporating accelerometer and objective gait balance, alongside symptom reporting, can also accurately predict in-clinic RA activity [20,21].

However, this level of patient input for RMT development and testing is uncommon. Multiple systematic reviews have highlighted the lack of patient involvement in the majority of the apps available, calling for more user experience research to feed into the design and development of app-based symptom measurement systems [12,22]. There is an even greater dearth of patient involvement in the development of platforms to merge subjective symptom reporting with passive data collection [23].

### Objectives

Accordingly, the aim of this body of research is to develop an app-based symptom measurement system that fully meets the needs of the patient and clinician users. This study represents the first step in this program, which is to elicit the views of patients to start developing the requirements for a system that captures both subjective and objective symptoms through patient-reported assessments and data collected via passive sensors. The objectives of this project are (1) to identify the symptoms prioritized by patients with RA for inclusion in a multiparametric RMT data collection platform and (2) to identify the key requirements that the platform would need to have for maximized utility, uptake, and long-term engagement with symptom management.

## Methods

### Design and Ethics

This study used qualitative methodology to understand patient views on the aspects of RA health and clinical care which may be most amenable to measurement via digital technologies. Semistructured focus groups explored the key symptoms and key requirements of an RMT platform. A topic guide (Multimedia Appendix 1) was developed based on recent systematic review evidence [16], which provided a loose framework for discussion. The focus groups were comoderated by a service-user researcher, experienced in qualitative research methods (RW), and the lead investigator (FM). Neither of the moderators were involved in patient care.

The study protocol and topic guide were approved by the Office for Research Ethics Committees Northern Ireland (ORECNI, REC number: 17/NI/0179).

### Study Participants and Recruitment

The eligibility criteria for inclusion in this study were as follows: (1) clinically verified RA, (2) aged 18 years or older, (3) able to speak English fluently, and (4) able to give informed consent. Patients were considered ineligible if they were unable to physically attend a focus group discussion at King’s College Hospital or had major cognitive impairment or dementia.

Eligible patients attending outpatient appointments at the King’s College Hospital National Health Service (NHS) Foundation Trust rheumatology service were consecutively approached and invited to participate. Patients were initially approached by a clinical trials practitioner, who was not directly involved in the patients’ clinical care. Patients were provided with an information sheet and provided verbal information about the nature of the project. With the patient’s consent, their contact details were provided to the lead researcher (FM), who then approached participants separately to discuss the study in detail.

All participants provided written informed consent to participate and were informed that their data would be anonymized and that they were free to withdraw at any time with no consequences to their clinical care.

### Data Collection

Two semistructured focus groups were conducted in November 2017 and January 2018. Both were conducted by two facilitators: facilitator 1 (RW), a lived-experience service user, and facilitator 2 (FM), a postdoctoral researcher and health psychologist at King’s College London. Each focus group lasted approximately 60 min, and participants also completed brief assessments to establish key demographics such as age, gender, comorbidities, and RA disease duration.

### Data Analysis

The focus groups were recorded and transcribed verbatim. Each participant was anonymized by assigning a unique number throughout the transcripts. Data-driven thematic analysis was used by two researchers (KW and AI), who independently analyzed both transcripts using NVivo 12 software (QSR International). Codes were discussed between the two researchers and grouped into overall themes. Owing to the data-driven approach used, it was anticipated that topics would emerge that deviated from the original interview guide. These were also included in the thematic analysis to highlight the concepts that the research team may not have considered in advance.

## Results

### Participant Characteristics

**Figure 1:**
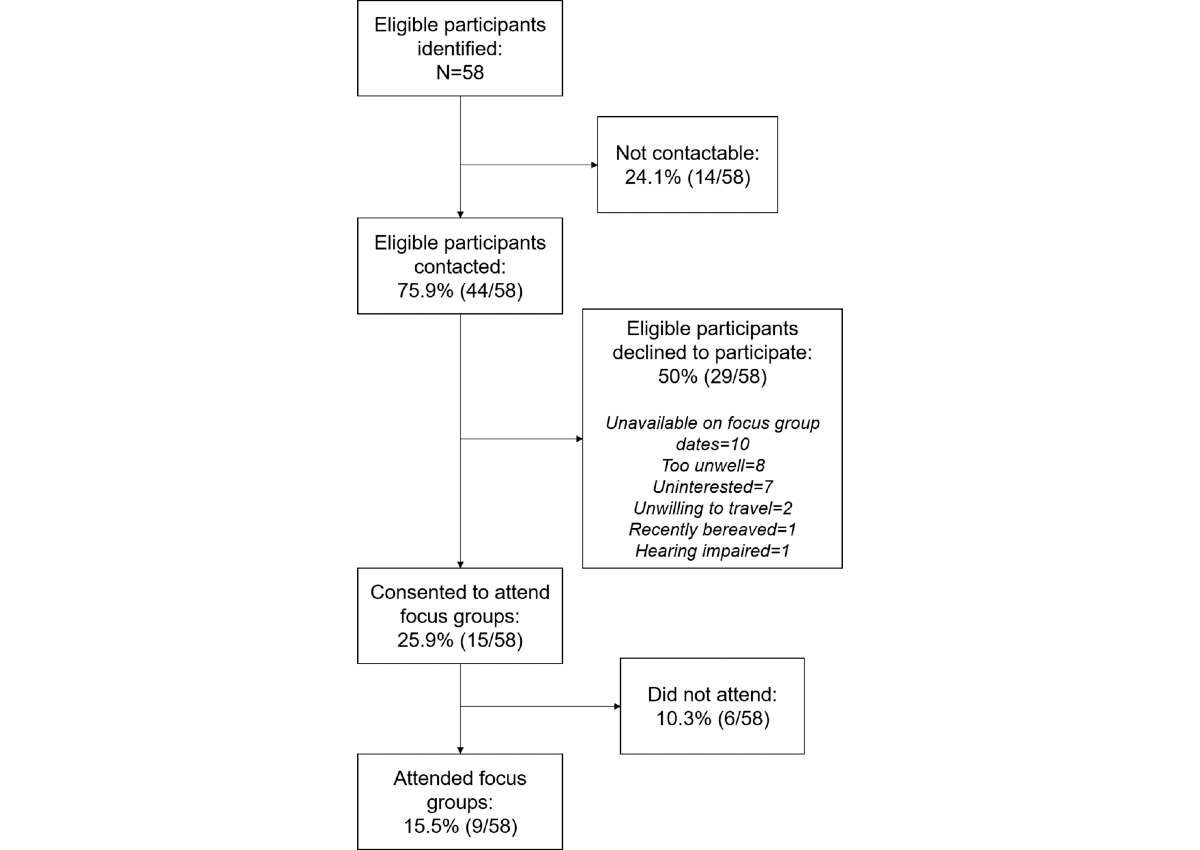
Participant flowchart. Percentages were calculated using the eligible participants identified (N=58) as the denominator.

Figure 1 shows the flow of participants from identifying eligible participants to the final sample group of 9. Table 1 shows final participant demographics. Participants were all females (N=9), aged 23 years to 77 years (mean 55.78, SD 17.54) who had lived with a diagnosis of RA for 1-47 years (mean 20.22, SD 14.33; Table 1). Ethnicities included White (n=6), White and Black African (n=1), Pakistani (n=1), and Caribbean (n=1).

**Table 1:** Participant demographics.

Participant number		Gender	Age (years), range	Ethnicity
**Focus group 1 (n=6)**				
	P1	Female	75-84	White other
	P2	Female	65-74	Pakistani
	P3	Female	35-44	White and Black African
	P4	Female	65-74	White British
	P5	Female	55-64	White British
	P6	Female	18-24	White British
**Focus group 2 (n=3)**				
	P7	Female	35-44	Caribbean
	P8	Female	65-74	White British
	P9	Female	45-54	White British

### Themes

A total of four overarching themes emerged from these data. These include the following: (1) important symptoms and outcomes in RA, (2) ways in which patients manage RA symptoms, (3) views on the current RA health care system, and (4) views on remote measurement in RA.

#### Symptoms Experienced and Important Outcomes

Patients with RA experience a host of symptoms related to both their physical and mental health. Both groups revealed pain and mobility to be the most important outcomes to assess and target during treatment:

You say you were crawling. That’s pain and stiffness…

Facilitator 1

Oh total pain, total pain.

P4

Well I agree with that. Well I always sort of say what I’m most scared of is losing my functioning, not the pain.

P8

Following this, mood was also seen as an important outcome. Patients explained how their physical symptoms impacted their mental health:

For me, to be as mobile as possible, so it’s not impinging on their lives really, and to control the pain, so I don’t get so irritable [chuckles]

P9

Physically, you’re also at a low ebb emotionally…because your body hurts. When you’re in pain, you try and keep your pecker up, but actually…

P4

Further discussions showed that the onset of RA had an impact not only on people’s health but also on their lifestyle. Important outcomes here related to maintaining social life and the ability to work and/or be a parent:

I gained my teaching assistant qualification July 2015 and then my health deteriorated the September, just after I got offered a job, so I had to turn the job down... I’m sitting there in agony with my feet, with my wrists, not being - having my wrists splintered, not being able to write at the board because my wrists are hurting so much.

P6

The inability to maintain a professional work life was also linked with the outcome of mood, with the same patient stating:

Yeah, even when I was diagnosed, first diagnosed, I was off work for four months. Initially, it’s like, yeah, I can relax, but then it’s, I don’t know what to do with myself. Then I know, personally, I’ll go down. I’m under the psychologist here as well.

P6

Although there was a general acknowledgment of the importance of these health and lifestyle outcomes in RA management, there were some individual differences between patients. They explained that these personalized impacts of RA are often the outcomes that are overlooked in the medical profession:

You know the outcomes about one’s life, is what matters. And that’s not always raised.

P8

#### Symptom Management

Symptom management emerged as the second key point of discussion. Ways of dealing with symptoms were first discussed in terms of medication and then in relation to the use of alternative symptom management strategies.

There was a general sense of uncertainty surrounding the effectiveness of medication in both focus groups. Some patients expressed positive experiences of using RA medication to improve outcomes, whereas others described medication as having had no effect on their symptoms. A couple of patients expressed reluctance to use traditional disease-modifying antirheumatic drugs (DMARDs) and steroid medications because of their effectiveness and side effects, respectively. They also felt that there was a lack of support in dealing with the confusion or fear surrounding taking different medications:

There’s nobody you can talk to, and you can’t talk to the consultants about it because it’s take it or leave it to some extent. But, there’s no experience to draw on, you know, have people got cancer from it, have they got septicaemia? What are the symptoms?

P9

Complementary treatments emerged as more acceptable than just taking medication alone. Patients reported improved outcomes from dietary changes, meditation, physiotherapy, and exercise. There was agreement that integration of medication and complementary treatments was important, with suggestions for a more holistic approach to RA management. Several barriers to this approach were highlighted, one being the perceived reluctance of health professionals to prescribe alternatives:

There seems to be a slight irritability if you’re reluctant to take the drugs from the doctors. And to explain that, you know, I’ve got 2 children, I don’t want to get cancer, I don’t want to have septicemia.

P9

they [consultant] said that there was no evidence to suggest that anything I was taking was going to help.... I was literally just told that my alternatives were not going to do anything for me, but I still went ahead.

P7

Other barriers to incorporating complementary treatments into RA management include the allocation of services based on location and restricted access to information. One patient suggested the creation of a website that personalizes exercise class availability based on location. That said, patients also tended to agree that pain or feelings of self-consciousness presented the biggest barrier to using exercise:

I think it’s that sort of encouragement because it’s a hard thing to do, particularly if you’re in pain. And also if you feel like your body isn’t right it makes you feel less good about your body or the idea of doing exercise.

P8

#### Current Views on the System and Ideas for Improvement

Key aspects in effective disease management include the importance of continuity of care, patient-centered care, and the impact of symptom fluctuations on appointment effectiveness.

Continuity of care was highlighted several times as a major area of importance. Perceived lack of coordination, both between departments and external health care providers, was cited as a barrier to continuity of care. Patients discussed their differing experiences in facilitating the link between their consultant, pharmacist, and prescription delivery service:

…I wasn’t able to do [DMARDs] for two weeks because I didn’t have any medication... I had to try and explain that to them. I said, I’ve been off my [medication name] since middle of September. You only delivered four [DMARDs]… So missing medication through no fault of my own was not really- so in terms of outcomes, I’d quite like to get my medication, please [laughs].

P3

Following this, patient-centered care was also discussed as being of equal importance. Patients reported not seeing the same consultants at each stage of their treatment, meaning that information is often unnecessarily repeated. One individual explained how their experience with an unfamiliar registrar made them feel as though their longstanding *story* and experience of RA was devalued:

Yeah, sort of treating the patient as an individual as well isn’t is, there’s a sort of party line and a protocol, but who is this person sitting in front of you, what is their story? She wouldn’t have had to read all the notes just to see I had been diagnosed a long time ago and have been coming to the clinic for many years.

P8

More positive experiences tended to include times when medical professionals acknowledged the outcomes of importance that were specific to the patient:

He was brilliant, because I love- I like working and I like travelling. I was going to see my daughter in Australia and I took my mum. He came- he notified my GP practice, who came round, pulled out [a pint] in each knee and injected me... he would do it in anticipation of- because I was going to be at my daughter’s wedding or whatever it was... I was a person. I was not a number.

P4

Patients went on to suggest how patient-centered care could be improved. For some, this involved building trust with medical professionals; for others, being able to provide some background information before appointments would suffice:

I know that sometimes you feel if only they had a card to give to all the persons before, if the person could report and that they could even tick, maybe, things so that, yes, that person has that or that or that- that would make it much easier for them.

P1

The third key issue questioned the effectiveness of current appointments. Both focus groups consistently noted that symptom fluctuations had a major impact on whether their appointments were effective. In general, it was agreed that symptom fluctuations, especially in relation to pain, are often not captured in appointments:

... when they say, how are you? Very often [laughs], if I go, sometimes I have no pain at that time... I forget that I had the pain because I tend to forget I’ve got pain, but then at times it’s, ooh [laughs], excruciating.

P1

One patient gave an example of the time that their appointment coincided with a period of heightened pain, which resulted in hospitalization. However, this was described as an exception, rather than the rule:

P4: I was very lucky. I had a - I didn’t know I had, but I had - I felt I had massive pains everywhere. I happened to be seeing Mr. X or Dr X the next day. He just - he took - I had a blood test done and my CRP was 300 and something and it should be below two... I was found a bed that - there and I was in until the Friday, when it was coming down...

P5: But that was luck more...

P4: Oh totally, totally.

Discussions surrounding how this could be improved were two-pronged. On the one hand, patients thought it a good idea to be able to conduct appointments or blood tests on days immediately after their pain symptoms. However, there was consensus that being able to log symptom flares for the purpose of reporting at the time of an appointment would be beneficial as well. It was even suggested that this could be in the form of a digitalized message, for example, a text or an email:

I wouldn’t mind getting an email saying, how have you been feeling this past week or month or even- that’s- I’d actually feel really quite positive about that.

P3

#### Remote Measurement in RA

Patients with RA might benefit both from providing personal information to new clinicians before appointments and keeping a log of symptom fluctuations to review during appointments. As such, each of the focus groups was asked about the potential of an RMT platform, such as a wearable and smartphone app, to track changes in their health.

Patients had varied views on which specific aspects of RA management should be tracked remotely. These generally culminate in two main symptoms: pain and mobility. With pain, patients were keen to highlight that pain can fluctuate by body part, as well as by time of day. With mobility, it was noted that this can vary both within and between days. These were all perceived as important aspects to capture in remote measurements:

I suppose I would imagine being able to sort of put in particular parts of the body which tend to have pain: joints, or in my case tendons as well. And then monitor maybe daily how they were doing. Some things that come and go, would be quite interesting.

P8

Maybe just the, maybe the periods of stiffness, when they are, is it different in the morning.

P9

One patient went as far as to describe a way in which their pain fluctuations could be visualized:

But if we had a - and we just ticked, for instance, with a timetable and ticked such a time that was crucial. You could even do it in color, I suppose. If it’s very bad, you could have it in red or black or green or whatever.

P1

It became apparent that it might be of interest to track not only the main symptoms but also the triggers of these symptoms. It was thought that this measurement might act towards preventing symptom flares. There was much deliberation over the symptom triggers that patients were already aware of, and these tended to differ by individual. In general, these included sleep, tiredness, diet, exercise, stress, mood, and other psychological factors. Often, these factors are present comorbidly, and it is difficult to determine the direction of causality:

Yeah I think maybe sleep would be another one. But it would be quite interesting to see how those things, sort of mood and also feelings of stress, and tension, how wound up you are about things, might see how that fluctuates, in parallel or not, with joint symptoms. And yes so, it works both ways. How it affects your sleep that’s actually quite a big one.

P8

Patients also thought it useful to have a list of common measures to track, alongside an *open box*, to note additional symptoms that are relevant to the individual:

Facilitator 2: Or if everyone gets to select before they even start what they want to measure, so if I could provide them with a long list of things that are available (voices saying yes) and before you even start you could say “I’m, I identify with this, this, this, and this”. And prioritize your top 5 things that you want to be able keep track of....

P8: Yes, that would make it more realistic, and even you know, you could review that after 6 months and change it, or look at different things for a period.

Although there seems to be a general sense that remote measurement in RA might be beneficial in some formats, there were a few differing views on whether encompassing this into an app would be helpful. This seemed to be dependent on the individual’s established coping mechanisms:

I’m an app freak, I’ve literally got an app for everything, anything around technology. So I thought, if there’s a way I can manage my arthritis through using an app then that would just be brilliant!

P7

The problem with the app, as we’re talking about it I think, is that the more you put in to sort of cover everything the more daunting it’s going to look like [laughs] the less everyone’s going to [interrupted]... it’s appealing but it all goes against my main coping strategy which is not thinking about it.

P8

## Discussion

### Principal Findings

**Figure 2:**
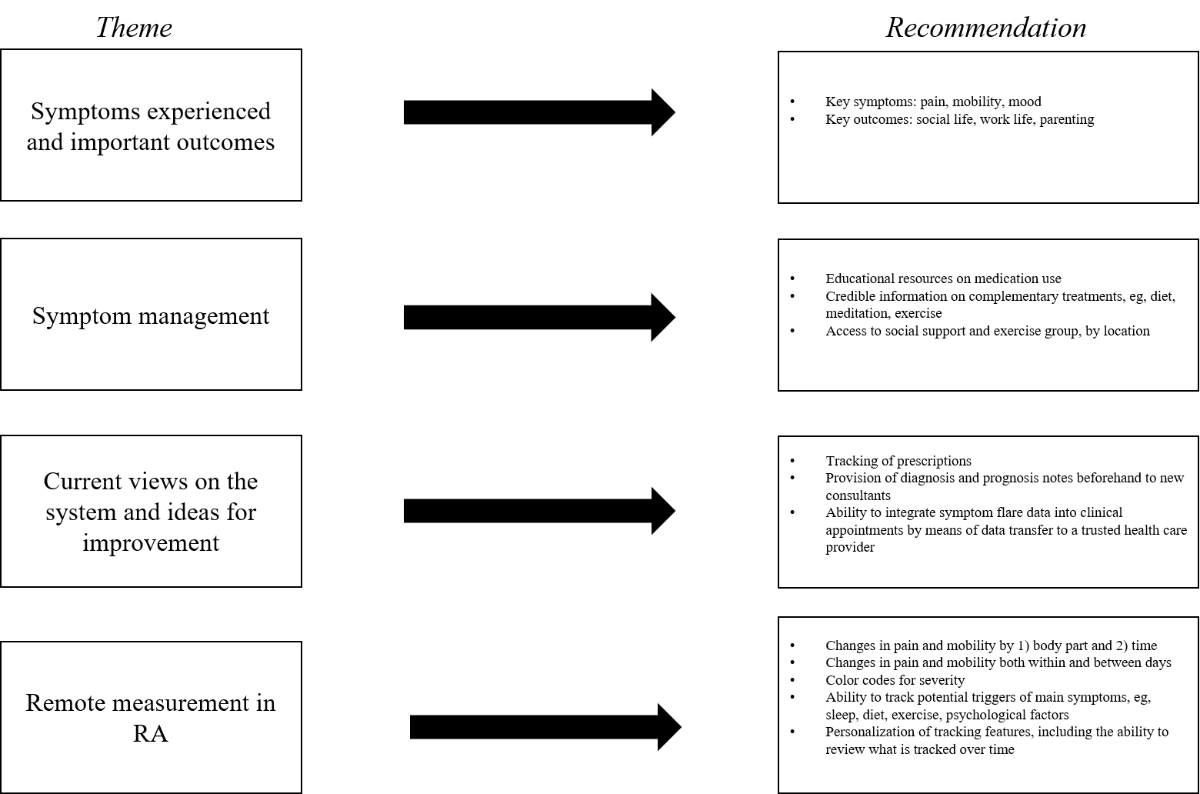
Key recommendations for consideration in a remote measurement technology system for rheumatoid arthritis symptom tracking, split by key theme. RA: rheumatoid arthritis.

This study aimed to provide the first step in developing a multiparametric RMT system for RA symptom tracking by eliciting views on (1) key symptoms prioritized by patients for inclusion in the system and (2) key requirements for maximized utility, uptake, and long-term engagement with RMT symptom management. In addition to these two original objectives, useful information about how RMT systems may be incorporated into existing health care services was elicited. A total of four key themes emerged: (1) key symptoms experienced and important outcomes to consider; (2) clinical and self-management strategies for RA symptoms; (3) ways in which the current health care system can be improved; and (4) facilitating RMT use in patients with RA. Figure 2 breaks down each of these themes into recommendations for consideration when designing a multiparametric RMT system for RA symptom tracking.

### Comparison With Previous Work

The results of this study align with previous findings supporting the acceptability of RMT apps for use in RA disease management [13-15,24]. Patients approved the development of an app for the purpose of individual symptom tracking. In terms of specific symptoms to include, results reinforce work led by Crouthamel et al [14] and El Miedany et al [19] in the admission that pain and mobility fluctuations are key symptoms to track. In particular, in the study by Crouthamel et al [14], use of a novel joint pain map for the interactive recording of the number and severity of painful joints seems particularly relevant. Our results suggest that patients might find it useful to be able to record these data at multiple periods throughout the day, alongside at weekly intervals. Interestingly, our results show that patients are also interested in tracking symptom triggers, such as sleep, diet, exercise, and mood. Reade et al [15] go some way toward capturing this in their study app, which collects self-reported measures across 10 variables, but crucially this work suggests that patients might prefer to personalize the symptom triggers they are reporting on, as well as to adapt these over time. Much of this work has focused on symptom tracking to improve health outcomes in patients with RA. Although our results suggest these to be of utmost importance, patients also placed value on lifestyle-related outcomes, such as work, parenthood, and socialization. It is less clear at this stage how these aspects of RA management would be incorporated into a symptom-tracking app.

In addition to tracking RA symptoms for self-management, these results suggest that an RMT system should also include a means of transferring data to a health care provider. Logistical barriers exist with respect to sharing data between third-party apps and secure NHS servers, so it is important to consider the value of linking patient symptom trackers with electronic health records (EHRs). Patients proposed the benefits of providing information before appointments, both for the purpose of aiding understanding of their condition and to accurately recall symptom fluctuations. This sentiment resonates well with qualitative work in this area [24]; participants were keen to use RMT tools to communicate with their physician, provided they felt that this would be incorporated into their care. In a similar sense, our findings suggest that provision of information to consultants seems dependent on the trust held for that professional. Previous work demonstrated that integrating remotely captured symptom fluctuations into EHRs presents an effective way for consultants to identify temporal changes and provide tailored disease management [18].

Our findings suggest that an app for patients with RA should include both symptom tracking and data transmission components. At present, the most popular apps available to download for RA management are those that combine symptom tracking and educational content [22]. Educational content, defined as information on disease pathology, diagnosis, or explanation of inputted symptom data, was not explicitly mentioned in our focus groups as an important feature of an app. However, discussions regarding disease management revealed uncertainty surrounding the use of RA medication and complementary treatments. An app that could provide dedicated information and recommendations for a range of treatment options might be the best fit. Interestingly, our results show that perceived reluctance to consider medication alternatives by health care providers is a barrier to treatment adherence. It might also be useful to consider combining educational content with data transmission features, such that patients could research their personal preferences and report back to consultants for review in upcoming appointments. In addition, patients reported motivation and access as key barriers to accessing complementary treatments. Inclusion of a social support feature in an app would go some way toward combating them. These results are in line with recommendations of Luo et al [22] to implement data transmission and access to social communities, alongside symptom tracking and education, into an app for RA management.

### Limitations

Several evaluation points must be considered. First, the sample size was small; therefore, additional themes related to this topic might have been missed, and there is limited generalizability to the wider RA population. Second, all patients across the two focus groups, although presenting a variation in age and disease experience, were female. Both facilitators were also women. Male participants were originally recruited but did not attend on the day. Including a female service user as a facilitator might have been more relatable to patients, yet results may still be biased toward a female experience of RA. It is not yet clear in the literature whether the perception of remote symptom measurement varies by gender. This is a common limitation, and the gender ratio is similar to other studies in the field [24]. Third, sampling for qualitative work inevitably results in a selection effect, whereby certain individuals might be more motivated to participate. Similarly, consecutive sampling through a single center excluded patients who were not currently attending outpatient appointments or could not physically attend a focus group. Given the fluctuations in pain and mobility that are apparent in RA, this might have skewed attendance toward those who were feeling well on the day. Had the groups included those presenting with symptom flares, certain themes may have been exacerbated. Fourth, the topic guide (Multimedia Appendix 1) was intentionally vague. This has elicited some important areas for discussion that were not previously considered by researchers, but it has also resulted in the underdevelopment of intended topics. For example, the concept of passive data monitoring has not been widely discussed in the groups, perhaps owing to the relative novelty of the technologies.

### Applications for Future Research

This work provides the foundation for developing a multiparametric RMT system for symptom management in RA. Having explored patient views on key symptoms and concepts for consideration, there are some clear applications for app design (Figure 2). An RMT system should include, at the very least, options to track changes in pain and mobility. Symptom severity may be best tracked visually, via a color chart, with the option of tracking changes by body part and over time. Ideally, users should also be able to add additional personalized symptoms and symptom triggers that they feel are pertinent to their experience of RA. There should also be some informational content available through the app regarding the use of medication or the local availability of complementary therapies. Developers should also consider the concept of data transmission of such information to relevant health care providers.

The next steps for future work in this body of research are to develop an RMT system that can undergo subsequent user testing with a similar group of patients with RA. Service-user workshops offer opportunities to facilitate the co-design of aspects such as user interface and usability [18]. Alongside app development, this should also include the provision of a passive, wearable device that can complement active symptom tracking. Given that our discussions found mobility to be a key outcome of importance, passive monitoring is likely to offer additional, unobtrusive insight into symptoms. These sessions could run over several days and include the option of attendance via video call to allow the inclusion of participants experiencing symptom flares or not attending clinic on the day. The purpose of such user testing should be to assess the usability and feasibility of the system and to understand how to maximize the utility of the data collected while minimizing the burden on patients. In turn, this would provide further insight into the barriers of uptake and long-term engagement with using RMT for symptom tracking.

A key requirement for implementing such technologies in clinical care is to assess the viewpoints of all stakeholders [10]. Parallel work should, of course, look to incorporate the views of rheumatology professionals into these discussions. This is especially relevant given that our findings highlight a clear desire to use an RMT system to send information to clinicians ahead of appointments, in the form of personalized details and symptom fluctuations. It is of upmost importance to assess the feasibility of such data transmission. This work could encompass a combination of both patient and clinician stakeholder views in single focus group sessions, discussing how the data would be incorporated into appointments.

### Conclusions

This paper has provided an in-depth exploration of the clinical outcomes valued by a group of patients with RA and, as a result, the key areas of consideration for inclusion in a disease management system. Static time point assessments miss important information for patients with fluctuating disease symptoms. Patients are interested in symptom tracking, and there is a clear and consistent message from patients that remote monitoring via an RMT system has a place in RA self-management and clinical care. This work has helped pave the way for the initial design of such an app, the success of which will be contingent on further co-design with patients. It should capture relevant and personalized outcomes with the possibility of integration with EHRs. Future work in this program aims to combine this app with passive symptom monitoring to create an optimal RMT system for RA symptom tracking. In the current climate of the COVID-19 pandemic, health services are witnessing a rapid shift toward remote management of disorders through telemedicine [25]. This work represents a step toward creating an acceptable and engaging remote system for use as an interface between self-management and clinical care during unprecedented times and beyond.

## Appendix

Multimedia Appendix 1 Semistructured focus group topic guide.

## Abbreviations

DMARD: disease-modifying antirheumatic drug
EHR: electronic health record
NHS: National Health Service
NIHR: National Institute for Health Research
RA: rheumatoid arthritis
REMORA: Remote Monitoring of Rheumatoid Arthritis
RMT: remote measurement technology
