# Supplementary material for: Remote Measurement in Rheumatoid Arthritis: Qualitative Analysis of Patient Perspectives
Source: JMIR Form Res. 2021 Mar 9;5(3):e22473. doi: 10.2196/22473 (PMC7988394; doi:10.2196/22473)
Supplement: Multimedia Appendix 1 [file formative_v5i3e22473_app1.docx]

# Topic Guide^[[1]](#footnote-1)^

## Focus Group

### Introduction (6.00-6.10pm)

- Introduce group facilitators, purpose of the project and layout of two focus group sessions
- Introduction to venue – fire escapes, toilet facilities, coffee/tea and refreshments.
- Introduce recording equipment and confirm ground rules for groups.
- Consent procedures: confirm information sheet contents and sign consent forms.
- Introduce group members: each person introduces themselves and describes their journey to the focus group venue.
- Brief Introduction to RADAR and concept of outcome measurement.

### Main discussion (6.10-7.20pm)

Objective 1 (6.10-6.40pm): understand the important outcomes valued by RA patients undergoing rheumatological pharmacotherapy.

- 1. When you last started a new treatment for your RA, or were first diagnosed, what were the outcomes you hoped to achieve?
     1. Why is this outcome important to you?
     2. You’ve mentioned X, is there anything else relating to your physical or mental health, lifestyle, RA management or relationships you would like to achieve?
  2. In the past, when you have had hospital or GP appointments for your RA, can you remember having any outcomes measured? If so, what outcomes?
     1. Is there anything your GP or rheumatologist measures regularly which you think is unimportant?
     2. Is there anything they currently do not measure which you think they should?
  3. Of all the different outcomes we’ve discussed as being important to you, which would be the most important to you as an individual?
     1. Try to come to a group consensus on what the priority would be.

Note: if participants are uncertain about what outcome means, clarify that we mean a consequence of treatment or diagnosis, or a goal that is worked towards either personally or with healthcare professional.

*Comfort Break (6.40-6.50pm)*

Objective 2 (6.50-7.20pm) to test the usability of an existing remote measurement platform and smartphone app to understand the changes required to maximise utility in RA patients.

- 1. When talked earlier, we discussed X, Y and Z as being important symptoms for you to understand and measure. Have you ever used a wearable or mobile technology to ever measure these types of things?
     1. Is there anything else you use technology to measure regularly?
     2. Any apps, inbuilt mobile phone sensors? Daily diaries? Alerts/notifications
     3. Wearable technology – activity/sleep monitoring.
  2. How would you feel about using a wearable or mobile technology to predict changes in your health?
     1. What motivations and/or do you have?
     2. Involvement of clinical care team?

### Close (7.20-7.30pm)

- Thank the group for their contribution
- Explain what will happen next
- Reminder of confidentiality
- Confirm date of second focus group

1. please note times are provided as a guide only and will vary depending on the specific time at which the focus groups are organised [↑](#footnote-ref-1)
